# Size-Controllable Melt-Electrospun Polycaprolactone (PCL) Fibers with a Sodium Chloride Additive

**DOI:** 10.3390/polym11111768

**Published:** 2019-10-27

**Authors:** Piyawat Piyasin, Rattakarn Yensano, Supree Pinitsoontorn

**Affiliations:** 1 Department of Physics, Faculty of Science, Khon Kaen University, Khon Kaen 40002, Thailand; pi.piyasil@gmail.com (P.P.); rattyen@kku.ac.th (R.Y.); 2 Institute of Nanomaterials Research and Innovation for Energy (IN-RIE), Research Network of NANOTEC-KKU (RNN), Khon Kaen University, Khon Kaen 40002, Thailand

**Keywords:** melt-electrospinning, fiber, polycaprolactone (PCL), additive, size

## Abstract

Melt-electrospun polycaprolactone (PCL) fibers were fabricated by using NaCl as an additive. The size and morphology of the PCL fibers could be controlled by varying the concentration of the additive. The smallest size of the fibers (2.67 ± 0.57) µm was found in the sample with 8 wt% NaCl, which was an order of magnitude smaller than the PCL fibers without the additive. The melt-electrospun fibers were characterized using the differential scanning calorimeter (DSC), X-ray diffraction (XRD), and Fourier transform infrared spectroscopy (FTIR) techniques. Interestingly, a trace of NaCl was not found in any melt-electrospun fiber. The remaining PCL after melt-electrospinning was evaporated by annealing, and the NaCl residual was found in the glass syringe. The result confirmed that the NaCl additive was not ejected from the glass syringe in the melt-electrospinning process. Instead, the NaCl additive changed the viscosity and the polarization of the molten polymer. Two parameters are crucial in determining the size and morphology of the electrospun fibers. The higher NaCl concentration could lead to higher polarization of the polymer melt and thus a stronger electrostatic force, but it could also result in an exceedingly high viscosity for melt-electrospinning. In addition, the absence of NaCl in the melt-electrospun PCL fibers is advantageous. The fibers need not be cleaned to remove additives and can be directly exploited in applications, such as tissue engineering or wound dressing.

## 1. Introduction

Micro/nano fibers possess several amazing properties, such as a high surface-to-volume ratio, high porosity, and flexibility [1]. Electrospinning is one of the techniques for fabricating micro/nano fibers via electrostatic force. This process can be divided into two sub-techniques: solution-electrospinning and melt-electrospinning [2]. Solution-electrospinning can produce polymeric fibers with a diameter ranging from tens of microns down to nanometers using various polymeric solutions [3,4]. However, the major challenges of solution-electrospinning are the potential environmental pollution from the solvent accumulation and the residual solvent in the fibers [5]. These drawbacks are the main obstacles for the mass production or utilization of such fibers in biomedical applications. On the other hand, melt-electrospinning is an eco-friendly process that can fabricate non-toxic fibers from molten polymers. Although an additional heating unit is required, the electrospun fibers from molten polymers remove the problem of the potential residual toxic solvent. Such advantages provide opportunities to use the melt-electrospun fibers in biomedical applications, such as tissue engineering, wound dressings, drug deliveries, filtration, and textiles [6,7]. However, the diameters of the melt-electrospun fibers are generally large (tens of microns) [8] in comparison to the fibers produced from solution-electrospinning. This larger size, due to the larger resistivity and viscosity of molten polymers [9], could result in some inferior properties and thus limit the fiber’s applications.

Two schemes to control the morphology and size of the melt-electrospun fibers have been explored. The first scheme entails adjusting the processing parameters, such as the molecular weight of the polymers, and the temperature, voltage, and the distance of the collector. Lyons et al. revealed that the tacticity and molecular weight of polypropylene (PP) directly influenced the size of the electrospun fibers [10]. The PP with larger molecular weights, as well as atacticity, resulted in electrospun fibers with larger diameters. Dalton et al. investigated the morphology of the melt-electrospun fibers by adjusting the molecular weight of the block copolymers between poly(ethylene glycol) and poly(ε-caprolactone) and found that the molecular weight is the predominant factor in the quality of the fibers [11]. Moreover, spinning temperature is critical to producing sub-micron sized fibers. Zhou et al. found that when the spinning temperature is below the glass transition temperature, whipping of the jet is suppressed, leading to a larger fiber diameter [12].

The second scheme to control the morphology and size of the polymeric fibers is by introducing additives. For instance, the diameter of the melt-electrospun PP fiber was found to decrease from 35.6 µm to 0.84 µm by the addition of 1.5% Irgatec CR 76 (sterically hindered hydroxylamine ether) [13]. Furthermore, Nayak et al. successfully fabricated sub-micron sized PP fibers by incorporating two different types of conductive additives: sodium oleate and sodium chloride [14]. Moreover, Malakhov et al. reported that sodium stearate, sodium oleate, and sodium myristate were used to improve the property of polyamide-6 melt-electrospun fibers [15]. It was found that the introduction of the additives into the polymer melt led to a decrease in viscosity and an increase in electrical conductivity.

Polycaprolactone (PCL) is a thermoplastic with a relatively low melting point (~60 °C) and glass transition temperature (−60 °C). PCL fibers have shown great advantages, such as biodegradability and non-toxicity, which make them a biomaterial candidate for use in tissue engineering and wound dressings [16,17]. However, PCL has very poor solubility in non-toxic solvents, such as water or dimethylsulfoxide (DMSO) [18]. Therefore, melt-electrospinning is advantageous in producing PCL fibers. For example, Farrugia et al. demonstrated that PCL fibers fabricated from melt-electrospinning in a direct writing mode were used as the scaffolds for seeding human dermal fibroblasts [19]. Due to the high porosity and interconnectivity of the scaffolds, the fibroblast cells were infiltrated successfully throughout and underneath the scaffold. In another study, the PCL fibers were directly melt-electrospun onto a pork liver with a long gash [20]. The study on the adhesive power and the temperature of the deposited fibers showed that the melt-electrospun PCL fibers have potential as a wound-healing material. Moreover, Fuchs et al. fabricated PCL scaffolds by using melt-electrospinning writing and demonstrated their use in oral wound healing [21]. They found that the scaffolds showed good cytocompatibility and were optimized for cell attachment and cell growth. In addition, Lian and Meng reported a comparative study between melt-electrospun PCL fibers versus solution-electrospun PCL fibers for drug delivery application [22]. Their results showed that the PCL fibers from melt-electrospinning possessed a smoother surface and higher crystallinity, which led to a larger drug uptake and a slower drug release rate.

In this work, we fabricated PCL fibers by the melt-electrospinning technique and investigated the change in the size and morphology of the fibers by introducing a sodium chloride (NaCl) additive. The effect of the additive concentration was studied. The idea of NaCl loading in PCL is to optimize the viscosity and the polarization of the molten polymer, both of which are crucial parameters in controlling the size and morphology of PCL fibers. The thermal properties, crystal structure, and functional groups of the melt-electrospun PCL fibers were investigated. Furthermore, the underlying reasons for changes in the size and morphology of the fibers were discussed by studying the viscosity and electrical conductivity of the molten polymer. The possible explanations for the observed results were also discussed.

## 2. Materials and Methods

### 2.1. Materials

The polycaprolactone (PCL) used in this study was purchased from Sigma-Aldrich (Queenstown, Singapore). Pellet-like PCL possesses the following characteristics: an average *M*_W_ (molecular weight) of 69,000, an average *M*_N_ (number average molecular weight) of 45,000 g mol^−1^, and a melting point of around 60 °C. The sodium chloride (NaCl) with an average *M*_W_ of 58.44 g mol^−1^ was selected as the additive for PCL fiber fabrication.

### 2.2. Melt-Electrospinning System

**Figure 1:**
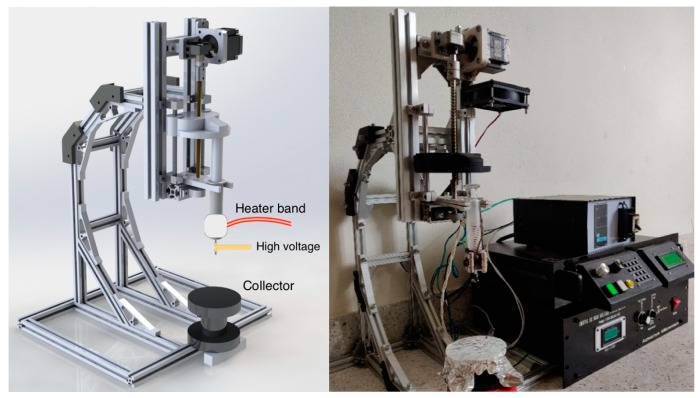
The melt-electrospinning apparatus used in this work.

The melt-electrospinning system used for fabricating PLC fibers in this study is a home-made apparatus, as shown in Figure 1. It consists of a high voltage supply, a syringe pump, a fiber collector and a heating system. The high voltage provides a maximum voltage output of 25 kV. The heating system is composed of a temperature controller, a band heater, and a thermocouple. The temperature controller (Shimax, MAC3B, Akita, Japan) was used to control the temperature (up to 400 °C) through a 190 W band heater, with a 24 mm inner diameter and a 30 mm length. A K-type thermocouple was used to monitor the temperature.

### 2.3. Fabrication of PCL Melt-Electrospun Fibers

In this work, the objective was to study the effect of an NaCl additive on the size and morphology of the melt-electrospun PCL fibers. Therefore, in the first step before the melt-electrospinning process, the PCL pellets were heated to 100 ± 5 °C to obtain the molten polymers. Then, various amounts of NaCl (up to 25 wt%) were added and vigorously stirred for 15 minutes to obtain a homogeneous solution, which was allowed to cool and solidify. After that, the NaCl-added PCL was loaded into a glass syringe and heated to 120 ± 2 °C and soaked for 10 minutes. In order to reduce the interference between the high voltage supply and the heating system, a negative voltage was applied to the nozzle of the glass syringe, and a positive voltage of 17 ± 0.1 kV was applied to the fiber collector. The fiber collector made from a plastic stub with a size of 8 cm × 8 cm was covered with aluminum foil and placed at 45 mm below the glass syringe. The molten polymer was pumped using a flow rate of 0.04 ± 0.01 mL/h through a stainless steel nozzle with a diameter of 0.7 mm.

### 2.4. Characterizations

The morphology of the melt-electrospun PCL fibers was observed by using a scanning electron microscope (SEM: SEC, SNE-4500M, California, USA). The fiber diameters were determined from the SEM images by averaging over 100 measurements using the Image J software (Wayne Rasband, version 1.52a, Maryland, USA). The thermal properties of the PCL fibers were measured using a differential scanning calorimeter (DSC: PerkinElmer, DSC 8000 Advanced Double-Furnace, Waltham, MA, USA). The samples were heated from room temperature to 100 °C with a heating rate of 10 °C/min and cooled to room temperature at the same rate. The crystalline structure of the fibers was studied by X-ray diffraction (XRD: PANalytical, EMPYREAN, Cambridge, UK). The samples were scanned with 2θ in the range of 5–80°. Fourier transform infrared spectroscopy (FTIR: Bruker, TENSOR27, Billerica, MA, USA) was used to investigate the functional groups of the PCL fibers in the wavenumber range of 400–4000 cm^−1^. Besides the fibers, the shear viscosity of the molten PCLs with and without NaCl was characterized by using a parallel plate rheometer (Physica, MCR 500, Cambridge, UK) in rotation mode. The molten PCL samples (25 mm in diameter and 1 mm thick) were analyzed over a wide shear rate (0.1–100 1/s) at a testing temperature of 120 °C and a strain of 3%.

## 3. Results and Discussion

**Figure 2:**
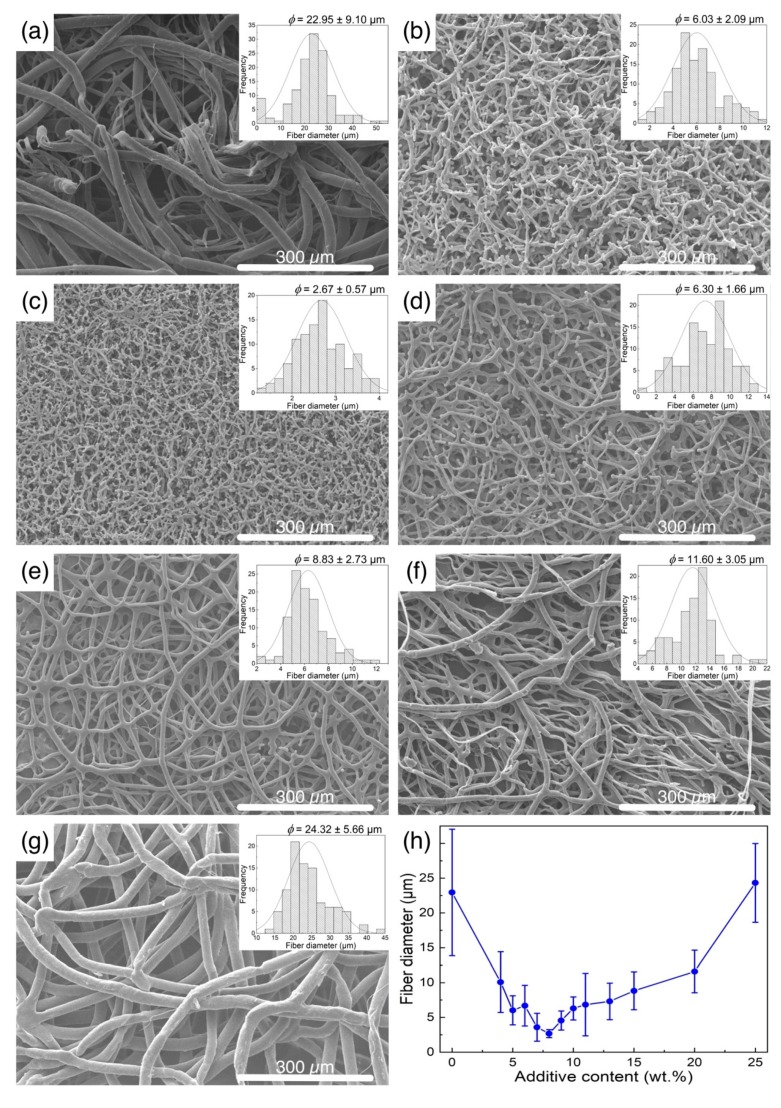
SEM images of the melt-electrospun Polycaprolactone (PCL) fibers: (**a**) without additive, (**b**–**g**) with an NaCl additive of (**b**) 5 wt %, (**c**) 8 wt %, (**d**) 10 wt %, (**e**) 15 wt %, (**f**) 20 wt %, and (**g**) 25 wt %. (**h**) Average diameter of the PCL fibers as a function of the additive content.

The surface morphologies of the melt-electrospun PCL fibers are shown in Figure 2, along with histograms for the fiber size and distribution. The continuous fibers with a smooth surface were found in the melt-electrospun PCL fibers without the additive, but a relatively large fiber (22.95 ± 9.10 µm) size was observed. On the other hand, the size and morphology of the NaCl-added PCL fibers significantly changed depending on the concentration of NaCl. For the PCL fibers with a small amount of NaCl (up to 8 wt%), the diameter of the fibers decreased with a larger amount of NaCl, but the surfaces were slightly rougher, and the continuity of the fibers was more disrupted. When the concentration of the additive was above 8 wt%, the surface and continuity of the melt-electrospun fibers became smoother and more continuous again, but with larger fiber diameters. The fiber size and distribution as a function of NaCl addition are shown in Figure 2h. The fiber diameters were reduced to the smallest value of (2.67 ± 0.57) µm at an additive content of 8 wt% and increased again for an additive concentration of 9 to 25 wt%. At 25 wt% NaCl, the average fiber diameter was about (24.32 ± 5.66) µm, similar to the PCL fiber without the additive.

**Figure 3:**
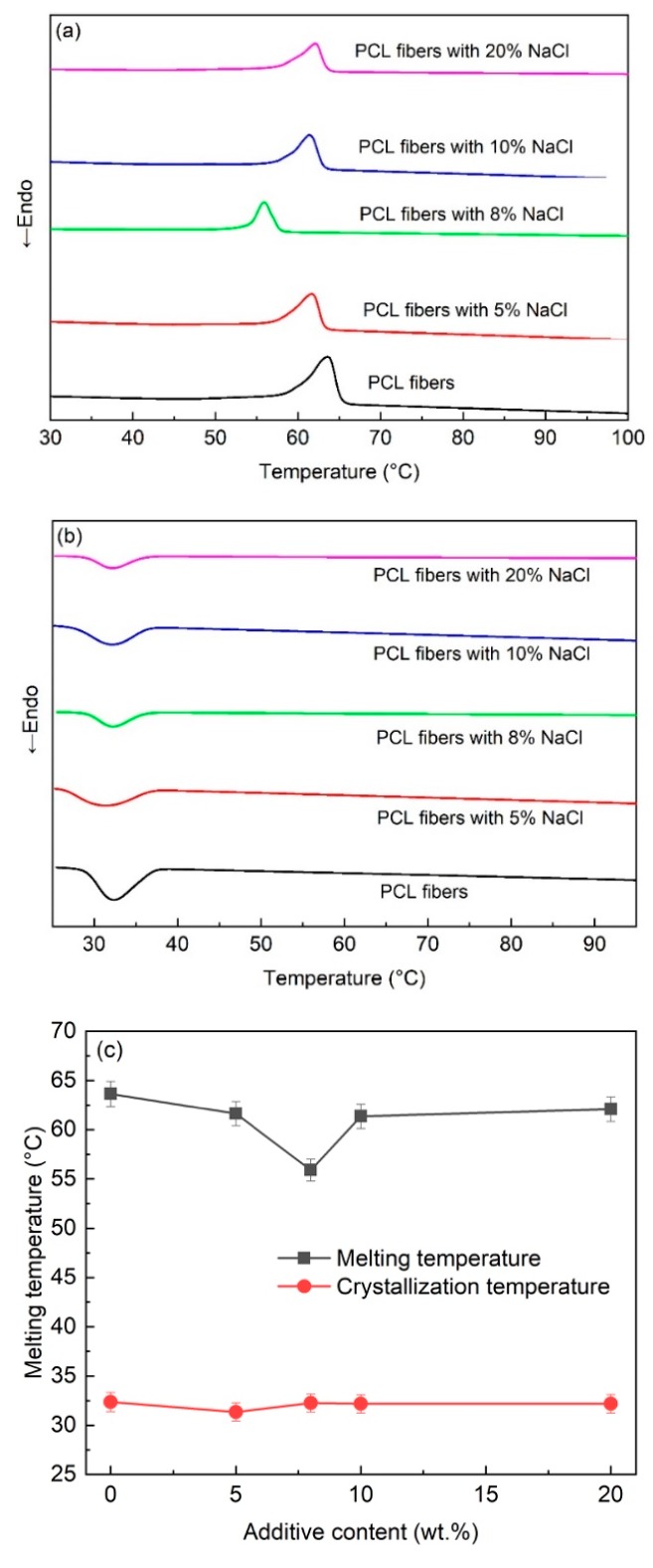
DSC thermograms of PCL fibers with different additive content: (**a**) heating and (**b**) cooling. (**c**) The melting and crystallization temperature of the PCL fibers derived from the DSC thermograms.

The thermal properties of the melt-electrospun PCL fibers were investigated using the DSC technique, as shown in Figure 3. The results showed that upon heating, the exothermic peaks for the melting temperature were observed in the range of 55–64 °C. The highest melting point was found for the PCL fibers without the additive, whereas the lowest melting point was observed for the PCL fiber with 8 wt% NaCl, as shown in Figure 3c. The change in the melting temperature follows the same trend as the change in size of the fibers. Therefore, the dependence of the melting temperature of the PCL fibers with the NaCl additive is attributed to the size of the fibers. The large fibers with lower surface areas led to a relatively high melting point, and vice versa. This finding is supported by a previous study of the electrospun polyethylene oxide (PEO) fibers, where a reduced melting temperature was observed with a decreasing fiber diameter [23].

Upon cooling, the DSC measurement showed the endothermic peaks for the crystallization of the PCL. Interestingly, the crystallization temperatures are relatively similar for all samples (~32 °C), independent of the additive’s concentration, which implies that the addition of NaCl did not affect the crystallization of the PCL after melt.

**Figure 4:**
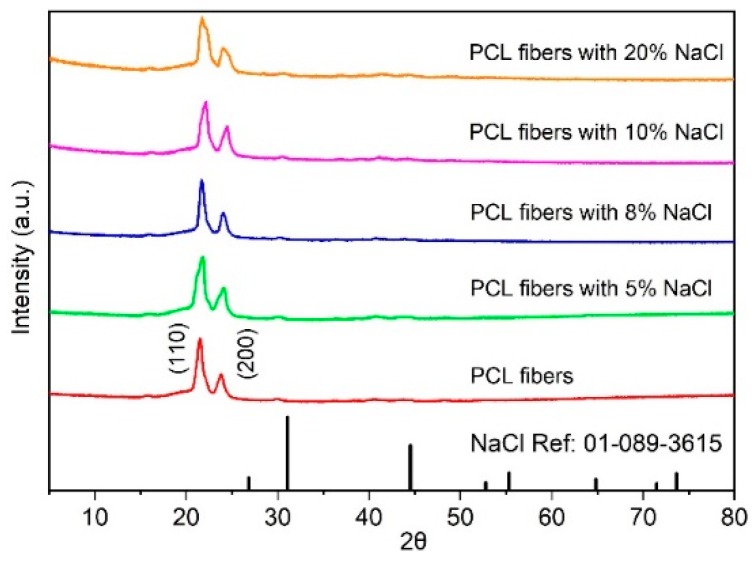
The XRD patterns of the PCL fiber and the PCL fiber with NaCl additive.

**Figure 5:**
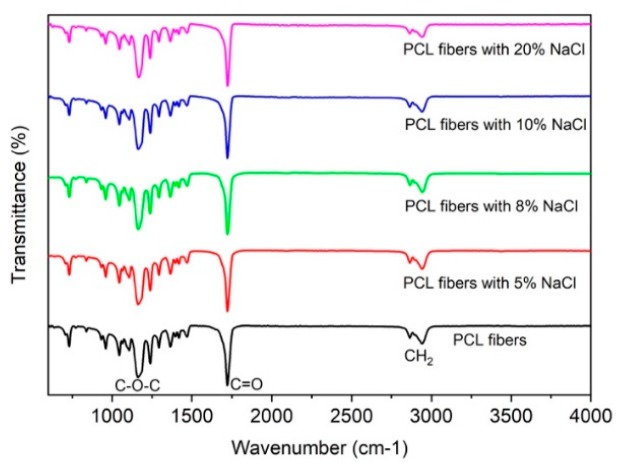
FTIR spectra of the PCL fiber and the PCL fiber with NaCl additives.

To further investigate why the NaCl additive affected the change in size and morphology of the melt-electrospun PCL fibers, an XRD and FTIR analysis was employed. As shown in Figure 4, the XRD patterns of the PCL fibers with and without NaCl additives are very similar. Both samples show diffracted peaks at 21.5° and 23.8°, corresponding to the (110) and (200) planes, respectively. The observed XRD peaks are similar to those previously reported for the electrospun PCL fibers [24]. Figure 5 shows the FTIR spectra of the PCL fibers with and without NaCl additive. Similar to the XRD results, no difference between the two samples can be observed. All samples showed the characteristic functional groups for PCL, such as a symmetric C–O–C vibration at 1171 cm^−1^, a C=O carbonyl group stretching at 1721 cm^−1^, an asymmetric CH_2_ stretching at 2943 cm^−1^, and a CH_2_ stretching at 2867 cm^−1^ [25]. Previous studies have shown the change in the FTIR spectra of the electrospun poly(l-lactide-*co*-glycolide) (PLGA) and polypropylene (PP) after sterilization [26], indicating the modification in the polymer’s structure and functional groups. However, this is not the case in our study—t here is no change in the FTIR spectra, which indicates that the addition of NaCl has no impact on the polymer structure and functional groups for PCL.

From the XRD and FTIR results, the evidence for the presence of NaCl in the melt-electrospun fibers was not observed. It is inferred that the NaCl additive was not ejected from the glass syringe during the melt-electrospinning process. This evidence is supported by the DSC analysis. If the NaCl is not present in the fibers, the composition of the fibers is presumably the same, independent of the use of the additive. Thus, upon heating, the melting temperature only depends on the size of the fibers. In other words, the smallest fibers with the largest surface areas exhibit the lowest temperature, which is precisely the observed results in Figure 3a. On the other hand, for cooling to room temperature, the polymer melts are exactly the same, leading to an insignificant difference in the crystallization temperature of all samples, as shown in Figure 3b.

**Figure 6:**
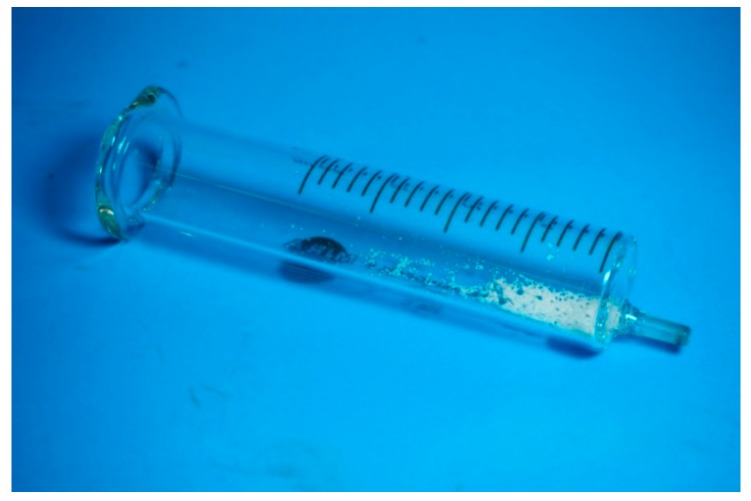
The remaining NaCl in the glass syringe after melt-electrospinning.

To confirm the findings from DSC, XRD, and FTIR, we collected the glass syringe right after the melt-electrospinning process with the remaining PCL inside. We placed the glass syringe in the oven at 400 °C for 2 h to remove the PCL. After annealing, we found the residual NaCl additive in the glass syringe (Figure 6). In this particular sample, the amount of the loaded NaCl in the polymer melt was 0.3309 g, whereas the measured weight of the NaCl residual was 0.3026 g (equivalent to 91.45% of the original weight). Hence, this result confirms that NaCl was not ejected through the nozzle during the electrospinning process. The measurement of the other samples yielded similar results. For the difference between the weight of the loaded NaCl and the weight of the residual NaCl in the glass syringe, a few explanations are possible. For example, when we stopped the melt-electrospinning process, there was still a drop of molten polymer through the needle before we could collect the glass syringe. This spillage could be one of the reasons for the measurement error. The other possibility is that the remaining NaCl was in the stainless-steel needle, which could not be recovered and accounted for in the weight measurement.

The observation of the NaCl in the glass syringe confirmed that the NaCl additive was not ejected through the nozzle in the melt-electrospinning process. Since the NaCl additive does directly affect effect the composition, functional groups, and crystallinity of the PCL fibers, it must have an effect on the polymer melt during the electrospinning process. In general, the viscosity of the molten polymers plays an important role in determining the size and morphology of the electrospun fibers. Sub-micron sized fibers could only be achieved in the range of the optimum visocosity. For example, the diameter of the melt-electrospun PP fibers was reduced from over 30 μm to 0.84 μm when the viscosity was decreased from 75 Pa·s to 33 Pa·s [11]. On the other hand, the size of the PEG_119_-*b*-PCL_189_ block copolymer fibers was reduced below 1 μm for the increased viscosity from 28.1 Pa·s to 59.5 Pa·s [9].

**Figure 7:**
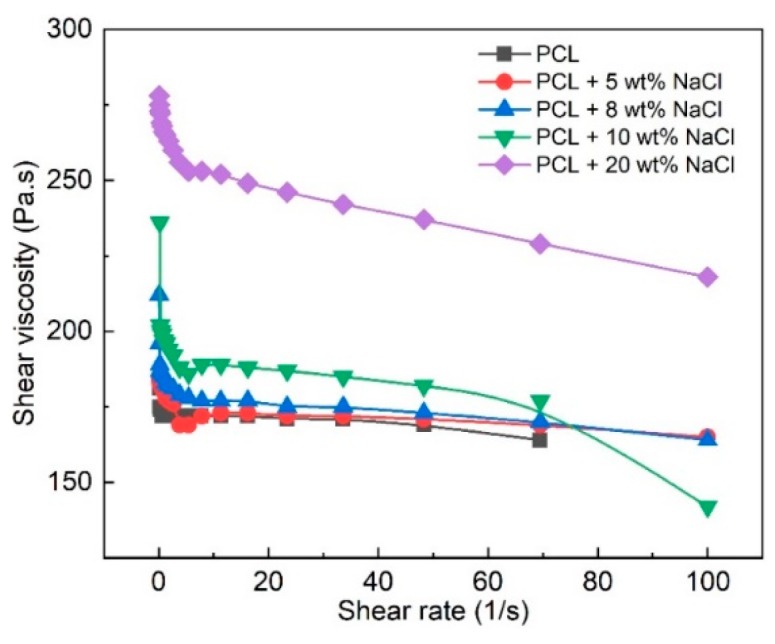
Shear viscosity of the PCL melt with different NaCl additive concentrations.

To determine the answer, we carried out a shear viscosity measurement of the molten PCL with different additive contents (0, 5, 8, 10, and 20 wt%), as shown in Figure 7. It can be observed that the addition of NaCl affected the viscosity of the molten polymer. The viscosity tended to increase following the increasing content of the additive. At 20 wt% of NaCl, the maximum shear viscosity was 278 Pa·s, which was about 54% higher compared to the maximum value (181 Pa·s) for the PCL melt without NaCl.

Another possibility for the NaCl additive is its influence on the electrical conductivity of the polymer melt. It was found previously that electrical conductivity is an important parameter that determines the charge density and attenuating force during electrospinning [14]. For instance, Cho et al. found that polyolefin in a decalin solution has an extremely low conductivity, leading to a difficulty electrospinning in the initial state [27]. Therefore, we have attempted to measure the electrical conductivity of the molten PCL using an impedance analyzer. However, the noise level was too high for the results to be compared reliably, which was due to a very low ionic conduction of the molten PCL from the limited solubility of NaCl in PCL. In addition, we have carried out a conductivity measurement of the melt-electrospun PCL fibers, but the results could not be obtained, possibly due to the non-conductive nature of PCL.

**Figure 8:**
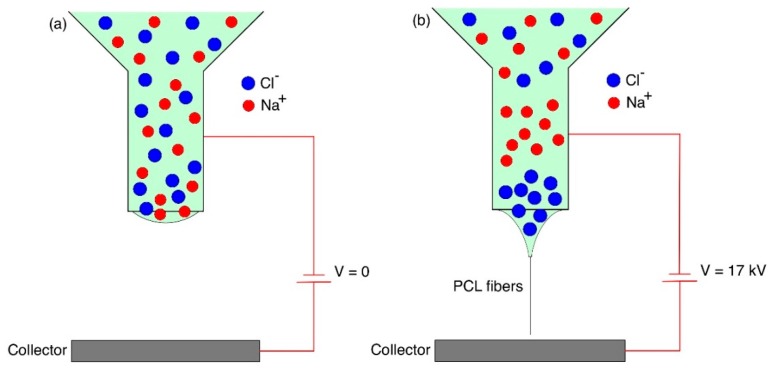
The schematic diagrams explain the dispersion of Na^+^ and Cl^−^ in the PCL melt when the high voltage is (**a**) off, (**b**) on.

Nevertheless, we can discuss the role of NaCl in the conductivity of the polymer melt based on the literature. Almost all polymers used in melt-electrospinning are not electrically conductive, which causes difficulties in fiber fabrication [9]. Chen et al. studied the effect of a polar additive (stearic acid) on the melt-electrospinning of PP fibers [28]. They measured the electrospinning current and found that the current increased with the amount of the added stearic acid. Similarly, our NaCl additive is also a polar material. In the NaCl-added PCL, the positive Na^+^ ions and negative Cl^−^ ions are dispersed in the molten polymer. If we do not supply a high voltage, the dispersion of Na^+^ and Cl^−^ ions are uniform in the molten polymer (Figure 8a). However, once the high voltage is switched on, the charged ions are attracted to the opposite poles, resulting in the increased ionic polarization of the PCL melt (Figure 8b). It should be noted that the polarization is very limited to the localized area and cannot form spatial polarization in wide areas. Therefore, the electrostatic force acting on the polymer melt, only at the proximity of the needle tip, is stronger for the PCL with the additive, and a smaller Taylor cone can be formed more easily. Since the parameters that could affect the fiber diameter and surface morphology, such as molecular weight, temperature, and electrical field [10,11,29] were controlled at the same conditions, the NaCl-added PCL was easily electrospun out of the end of the Taylor cone, with relatively smaller diameters compared to the polymer without the additive.

## 4. Conclusion

In this work, we have fabricated PCL fibers via the melt-electrospinning technique, and NaCl was used as an additive. It was found that the size and morphology of the PCL fibers depended on the concentration of the additive. The size of the PCL fibers decreased continuously with the NaCl concentration up to 8 wt% and increased again for the larger amount of NaCl. The smallest fiber diameter of (2.67 ± 0.57) µm was successfully fabricated for the PCL with 8 wt% NaCl. However, the results from DSC, XRD, and FTIR showed that NaCl does not exist in the PCL fibers. These results were confirmed by the evidence of the remaining NaCl residue in the glass syringe after melt-electrospinning and polymer removal. Hence, it can be concluded that during fiber solidification, the NaCl is driven out of the fiber volume due to the small thermodynamic solubility of the electrolyte, but in the polymer melt, this component is distributed in the melt bulk in a noticeable quantity that was confirmed by the variation in viscosity and fiber diameter. The shear viscosity was found to increase with NaCl concentration. Similarly, the polarization of the PCL melt was believed to increase with NaCl content. These two effects would significantly influence the electrospinning process since viscosity and polarization of the molten polymers are crucial parameters in determining the size and morphology of the electrospun fibers. Although adding a greater amount of NaCl could lead to higher polarization of the polymer melt, it also resulted in an inappropriately high viscosity for electrospinning. Therefore, the samples with 8 wt% NaCl are likely to exhibit the optimum viscosity and polarization of the molten polymer for the melt-electrospinning process.

The fact that the size of the PCL melt-electrospun fibers could be controlled using the NaCl additive, even though the additive does not exist in the fibers, is very useful, as the additive does not need to be cleaned afterward. Thus, native PCL fibers with a controllable size could be directly applied to tissue engineering or wound dressing applications.
